# Maternal low intensity physical exercise prevents obesity in offspring rats exposed to early overnutrition

**DOI:** 10.1038/s41598-017-07395-2

**Published:** 2017-08-09

**Authors:** Tatiane Aparecida Ribeiro, Laize Peron Tófolo, Isabela Peixoto Martins, Audrei Pavanello, Júlio Cezar de Oliveira, Kelly Valério Prates, Rosiane Aparecida Miranda, Claudinéia Conationi da Silva Franco, Rodrigo Mello Gomes, Flávio Andrade Francisco, Vander Silva Alves, Douglas Lopes de Almeida, Veridiana Mota Moreira, Kesia Palma-Rigo, Elaine Vieira, Gabriel Sergio Fabricio, Marcos Ricardo da Silva Rodrigues, Wilson Rinaldi, Ananda Malta, Paulo Cezar de Freitas Mathias

**Affiliations:** 1 Laboratory of Secretion Cell Biology, Department of Biotechnology, Genetics and Cell Biology, State University of Maringá, Maringá, 87020-900 PR Brazil; 2 Department of Physical Education, State University of Maringá, Maringá, PR Brazil; 3 Institute of Health Sciences, Federal University of Mato Grosso, Sinop, MT Brazil; 4 Laboratory of Molecular Endocrinology, Carlos Chagas Filho Biophysics Institute, Federal University of Rio de Janeiro, Rio de Janeiro, RJ Brazil; 5 Laboratory of Neuroscience and Cardiovascular Physiology, Department of Physiological Sciences, Federal University of Goiás, Goiânia, GO Brazil; 6 Program in Physical Education, Catholic University of Brasilia, Brasilia, Brazil; 7 Department of Medicine, State University of Ponta Grossa, Ponta Grossa, PR Brazil

## Abstract

Low intensity exercise during pregnancy and lactation may create a protective effect against the development of obesity in offspring exposed to overnutrition in early life. To test these hypotheses, pregnant rats were randomly assigned into 2 groups: Sedentary and Exercised, low intensity, on a rodent treadmill at 30% VO_2Max_ /30-minute/session/3x/week throughout pregnancy and the lactation. Male offspring were raised in small litters (SL, 3 pups/dam) and normal litters (NL, 9 pups/dam) as models of early overnutrition and normal feed, respectively. Exercised mothers showed low mesenteric fat pad stores and fasting glucose and improved glucose-insulin tolerance, VO_2max_ during lactation and sympathetic activity. Moreover, the breast milk contained elevated levels of insulin. In addition, SL of sedentary mothers presented metabolic dysfunction and glucose and insulin intolerance and were hyperglycemic and hyperinsulinemic in adulthood. SL of exercised mothers showed lower fat tissue accretion and improvements in glucose tolerance, insulin sensitivity, insulinemia and glycemia. The results suggest that maternal exercise during the perinatal period can have a possible reprogramming effect to prevent metabolic dysfunction in adult rat offspring exposed to early overnutrition, which may be associated with the improvement in maternal health caused by exercise.

## Introduction

Nutritional, hormonal and metabolic insults during early critical periods of life can predispose individuals to long-lasting deleterious effects later in life^1^. This phenomenon has been known as metabolic programming^2^. Studies have shown that poor or overnutrition during perinatal life is associated with an increased risk of type 2 diabetes and other chronic diseases later in life^3^.

A healthy lifestyle including a balanced diet and regular physical exercise during perinatal life can have positive effects on maternal metabolism and that of the subsequent generation^4, 5^. Physical exercise during pregnancy is known to have beneficial effects on maternal health, decreasing the risk of preeclampsia and gestational diabetes^6^. In addition, aerobic physical exercise in lactating woman improves maternal maximal oxygen consumption (VO_2max_) and plasma high-density lipoprotein (HDL) cholesterol concentrations^7^.

On the other hand, high intensity physical exercise during pregnancy in women can affect fetal health, inducing maternal hyperthermia^8^, increased uterine contractility by hormone stimulation^9^, fetal hypoglycemia^10^, and reduction in visceral and placental blood flow due to diverted blood to the working muscles mass and skin^11^. Higher intensity exercise over a long duration during pregnancy can induce negative outcomes in human and rodent offspring^12, 13^.

The current study highlights the scarcity of a clear recommendation regarding the type, timing, intensity, frequency and duration and benefits of exercise; therefore, we aim to evaluate whether maternal low intensity exercise during pregnancy and lactation can attenuate the adult metabolic dysfunction induced by early postnatal overnutrition in offspring rats.

## Results

### Effects of low intensity physical exercise during pregnancy and lactation on theVO_2max_ parameters of the dams

Table 1 shows the VO_2max_values in the dams. There was no difference in theVO_2max_ values before physical exercise between groups (Table 1). However, after physical exercise, at lactational day 3 (LD3), exercised mothers with normal litter (EM-NL) and exercised mothers with small litter (EM-SL), showed an increase of 12% and 20% in the VO_2max_ compared with sedentary mothers with normal litter (SM-NL) and sedentary mothers with small litter (SM-SL) respectively (*p*_*E*_ < *0.0001* – Table 1).

**Table 1:** Effect of low intensity physical exercise training on metabolism and fat pad stores in mothers.

Parameters	SM-NL	SM-SL	EM-NL	EM-SL	Source of variation
Fasting Insulin LD21 (ng/mL)	0.55 ± 0.05	0.58 ± 0.04	0.52 ± 0.07	0.57 ± 0.04	LxE^ns^ / E^ns^ / L^ns^
Fasting Glucose LD21(mg/dL)	99.5 ± 2.46	97.4 ± 2.56	79.5 ± 3.75	82.0 ± 4.76	LxE^*^ / E^***^ / L^ns^
HOMA-IR LD21	3.15 ± 0.46	2.88 ± 0.26	2.65 ± 0.42	2.96 ± 0.33	LxE ^ns^ / E^**^ /L^*^
Mesenteric fat pad LD21(g/100 g bw)	0.72 ± 0.03	0.75 ± 0.04	0.51 ± 0.006	0.57 ± 0.03	LxE^ns^ / E^****^/ L^ns^
VO_2max_ Pregnancy day 0.5 (mL/kg/min)	25.8 ± 1.01	25.13 ± 0,85	23.22 ± 1.23	22.45 ± 1.00	LxE ^ns^ / E^ns^/ L^ns^
VO_2max_ LD3 (mL/kg/min)	19.46 ± 0.95	18.56 ± 0.73	22.52 ± 0.36	22.73 ± 0.35	LxE^ns^/ E^****^/ L^ns^
Parasympathetic electrical activity LD21(spike/s)	16.04 ± 1.94	15.87 ± 1.49	16.79 ± 1.88	16.75 ± 1.84	LxE^ns^ / E^ns^/ L^ns^
Sympathetic electrical activity LD21(spike/s)	16.58 ± 1.06	17.04 ± 1.00	23.32 ± 2.32	25.19 ± 2.42	LxE^ns^ / E^***^/ L^ns^

Data are expressed as the mean ± SEM. at LD21 (n = 10–12 per group). LxE, interaction between the exercise factor and the litter factor; E, exercise factor and L, litter factor; *p < 0.05, **p < 0.01, ***p < 0.001, ****p < *0.0001* and ns, no significant difference, based on a two-way analysis of variance.

### Effects of low intensity physical exercise during pregnancy and lactation on the body composition of the dams

**Figure 1:**
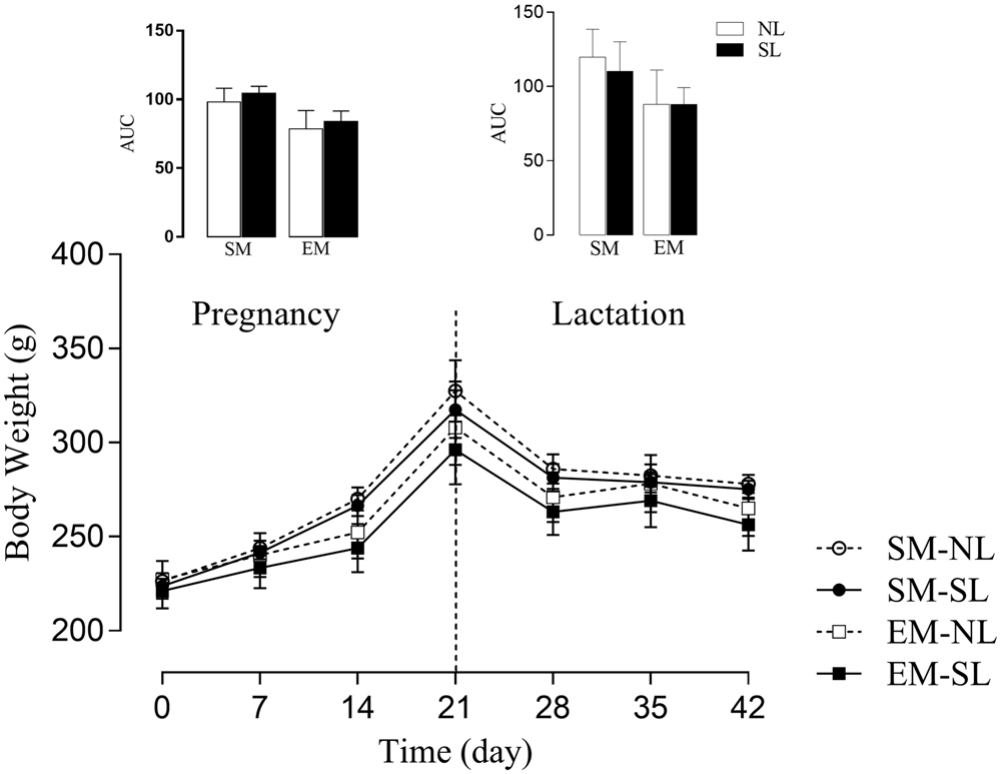
Body weight in mothers. Effect of low intensity physical exercise training on the body weight of the mothers. The upper panel represents the area under the curve (AUC) of bw during pregnancy and lactation (n = 10–12 per group). LxE, interaction between the exercise factor and the litter factor; E, exercise factor and L, litter factor; *p < *0.05*, each time points of the bw curve, was calculated by repeated measures ANOVA.

No difference was found in the maternal weight during pregnancy and lactation between the groups (Fig. 1). There were differences in maternal mesenteric fat pad stores in LD21, EM-NL and EM-SL, showing 23% and 24% decreased maternal mesenteric fat pad stores compared with SM-NL and SM-SL respectively (*p*_*E*_ < *0.0001* – Table 1).

### Effects of low intensity physical exercise during pregnancy and lactation on the milk and plasma biochemical parameters of the dams

**Figure 2:**
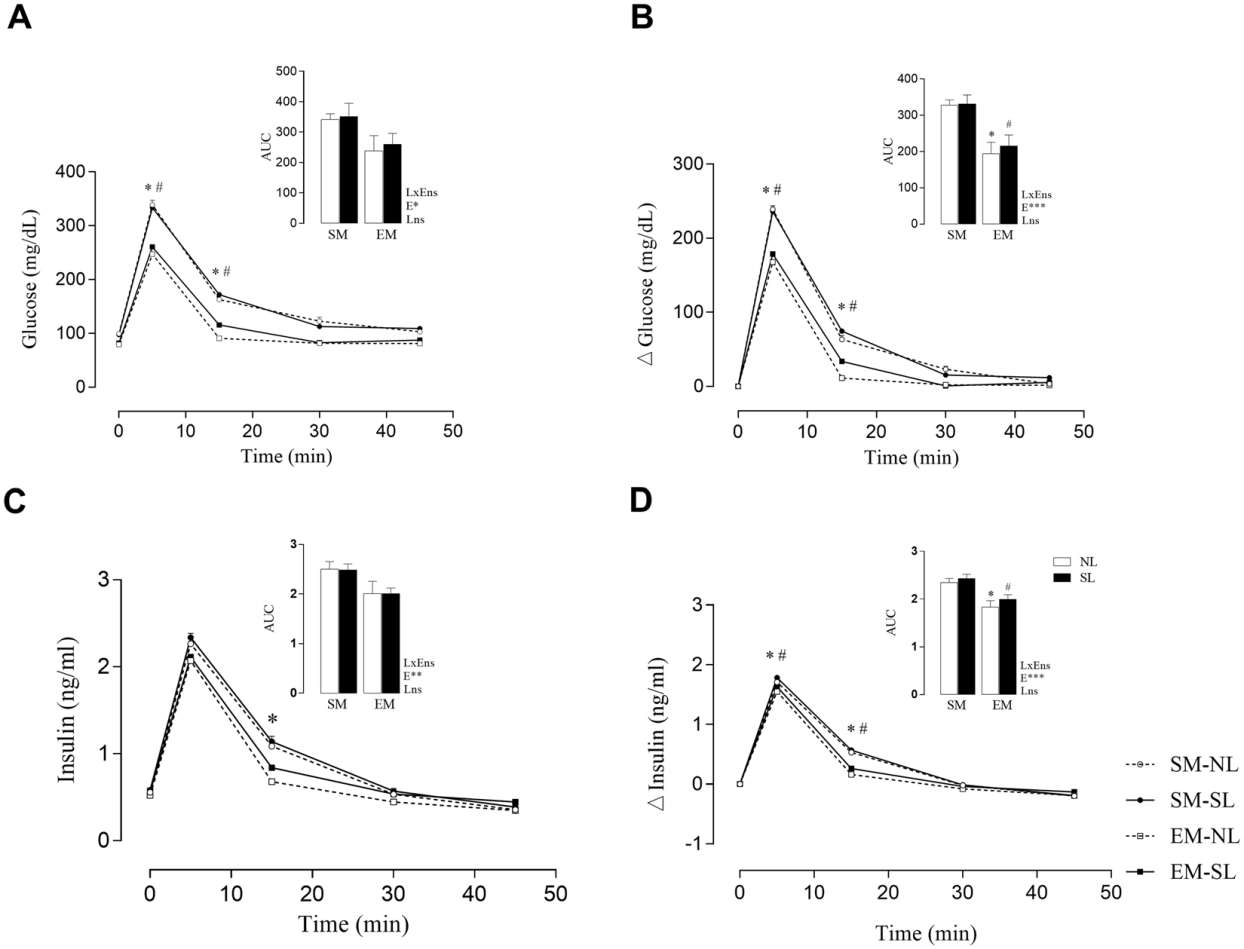
Effect of low intensity physical exercise training on glucose and insulin in mothers during the ivGTT. The upper panel represents the area under the curve (AUC). (**a**) plasma glucose, (**b**) plasma insulin,(**c**) increments (Δ) of plasma glucose and (**d**) increments (Δ) of plasma insulin after weaning until LD21 (n = 10–12 per group). LxE, interaction between the exercise factor and the litter factor; E, exercise factor and L, litter factor; *p < *0.05* and ***p < *0.001* by Student’s *t* test. And ****p < *0.0001*, each time points of the plasma glucose and insulin during ivGTT, was calculated by repeated measures ANOVA.

**Figure 3:**
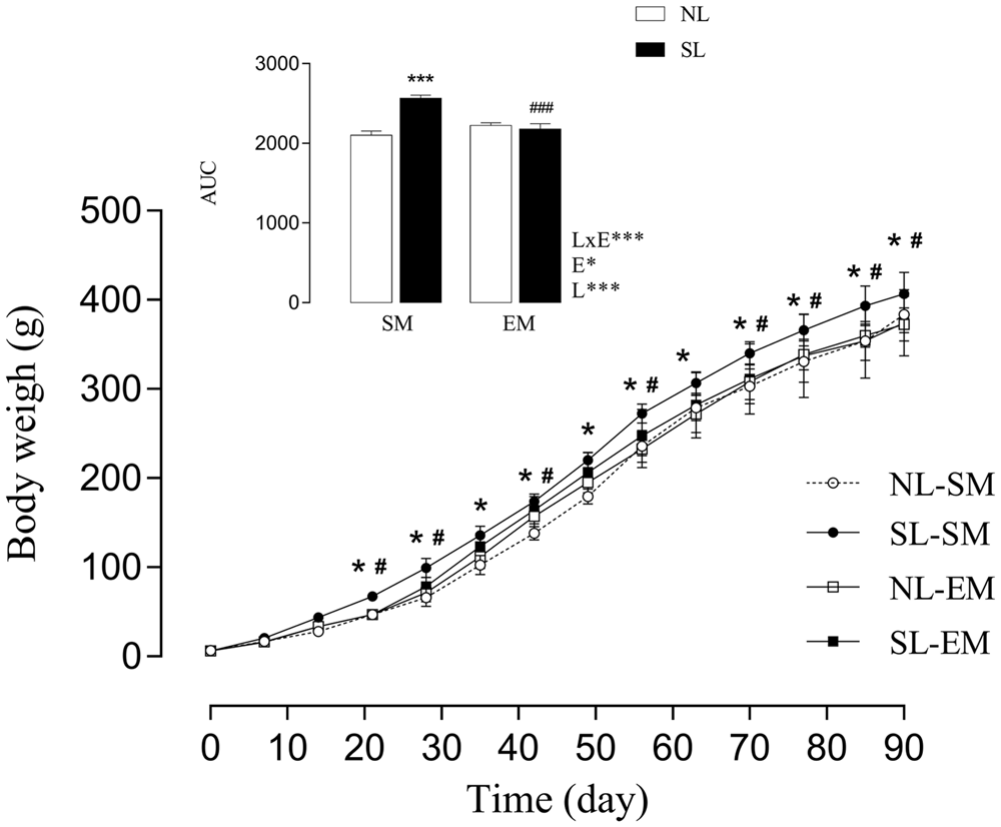
Effect of low intensity physical exercise training on adult offspring body weight. Area under the curve of the body weight. LxE, interaction between the exercise factor and the litter factor; E, exercise factor and L, litter factor; *p < *0.05* and ***p < *0.0001* by two-way ANOVA and Tukey’s test. (n = 6–18). The ^#^ or * represents *p* < *0.05* in each time points of the bw curve, was calculated by repeated measures ANOVA. Over the lines and bars, (*) represents NL-SM compared with SL-SM and (^#^) represents SL-SM compared to SL-EM.

Low intensity exercise in EM-NL and EM-SL, resulted in a lower fasting plasma glucose compared with SM-NL and SM-SL, by 18.2% and 16% respectively (*p*_*E*_ < *0.01* – Table 1), while plasma insulin levels were not different between the groups (Table 1). In Fig. 3a, during the ivGTT, EM-NL and EM-SL, plasma glucose, showed a significant lower difference at 5 peak time point of the plasma glucose, 27% (*p* < *0.0001*) and 44% (*p* < *0.001*) than SM-NL and SM-SL respectively, as well as at 15 peak time point, the plasma glucose was 21.8% (*p* < *0.001*) and 32.6% (*p* < *0.01*) lower in EM-NL and EM-SL than SM-NL and SM-SL respectively. Similar results were observed in plasma glucose increment during ivGTT in Fig. 2b.

The Fig. 2c shows that EM-NL present lower plasma insulin levels during ivGTT, at 15 peak time point, 33%(*p* < *0.01*) than SM-NL, as well as the Fig. 2d, shows a lower plasma insulin increment during ivGTT at 5 peak time point, 10% (*p* < *0.01*) and 8.4% (*p* < *0.05*) than SM-NL and SM-SL respectively. Likewise, at 15 peak time point, the plasma insulin increment was 71% (*p* < *0.0001*) and 56% (*p* < *0.0001*) lower in EM-NL and EM-SL than SM-NL and SM-SL respectively.

No difference was found in the HOMA-IR between the groups (Table 1). There was no difference in the glucose and lipid composition of the milk between the groups. However, total cholesterol content at day 21 was 10% and 20% lower in EM-NL and EM-SL, than SM-NL and SM-SL respectively (*p*_*E*_ < *0.05* – Table 2). Interestingly, EM-NL and EM-SL, exhibited an increase in insulin levels in the milk on the 10^th^ (58% and 62%) and 21^st^ (106% and 97%) day of lactation, compared to that of SM-NL and SM-SL respectively (*p* < *0.0001* – Table 2).

**Table 2:** Effect of low intensity physical exercise training on milk composition.

Parameters	SM-NL	SM-SL	EM-NL	EM-SL	Source of variation
**Day 10 of lactation**					
Insulin (ng/mL)	1,40 ± 0.07	1.24 ± 0.10	2.22 ± 0.17	2.01 ± 0.19	LxE^ns^ / E**** / L^ns^
Glucose (mg/dL)	139 ± 11.0	146 ± 10.4	142 ± 11.2	151 ± 11.0	LxE^ns^ / E^ns^/ Lns
Triglycerides (mg/dL)	1738 ± 176	2170 ± 584	1487 ± 117	1606 ± 181	LxE^ns^ / E^ns^/ Lns
Total Cholesterol (mg/dL)	8.53 ± 9.0	89.02 ± 8.54	79.71 ± 7.5	83.96 ± 8.3	LxE^ns^ / E^ns^/ Lns
**Day 21 of lactation**					
Insulin (ng/mL)	1.15 ± 0.15	1.25 ± 0.15	2.37 ± 0.58	2.46 ± 0.51	LxE^ns^ / E**** / L^ns^
Glucose (mg/dL)	200 ± 39	210 ± 35,2	189 ± 19	220 ± 23.8	LxE^ns^ / E^ns^/ Lns
Triglycerides (mg/dL)	3373 ± 826	3786 ± 715	2831 ± 363	3292 ± 351	LxE^ns^ / E^ns^/ Lns
Total Cholesterol (mg/dL)	143 ± 14.0	163 ± 5.0	128 ± 8.8	129 ± 9.3	LxE^ns^ / E^*^/ Lns

Data are expressed as the mean ± SEM. (n = 10-12 per group). LxE, interaction between the exercise factor and the litter factor; E, exercise factor and L, litter factor; *p < *0.05* and ****p < *0.0001* and ns, no significant difference, based on a two-way analysis of variance.

### Effects of low intensity physical exercise during pregnancy and lactation on the autonomic nervous system of the dams

We observed no difference in parasympathetic nervous system activity between the groups (Table 1). Nevertheless, the EM-NL and EM-SL, presented a 40.6% and 47.8% increase in sympathetic nervous system activity compared to the SM-NL and SM-SL respectively (*p* < *0.001* – Table 1).

### Long-term effects of low intensity physical exercise in pregnant and lactating dams on the body composition of the adult offspring

As observed in Table 3, there was no difference in birth weight between the offspring groups (*p* = *0.6*). Early overnutrition induced an increase in the bw of the rats in the SL-SM group at P21 and P90 of 44.6% (*p*_*l*_ < *0.001*) and 10% (*p*_*l*_ < *0.05*), respectively, compared to that of rats in the NL-SM group. The SL-SM group showed higher body weigh at P21 through P90, compared to that of the NL-SM, as well as, the SL-EM group showed low body weigh at P21 through P90, compared to that of the SL-SM (*p* < *0.05* - Fig. 3). In Fig. 3, the evolution of the body weight, as indicated that SL-SM showed a higher body weight since P21 until P90 (21, 28, 35, 42, 49, 56, 63, 70, 85, 90 days old) represented by the AUC, the SL-SM group was 22.1% higher than in the NL-SM rats (*p*_*l*_ < *0.001*). In contrast, the NL-EM group showed no difference in the bw curve compared to that of the NL-SM group. SL-EM rats had a lower body weight than the SL-SM rats, (at 21, 28, 42, 56, 70, 85, 90 days old) resulting in a significant interaction between litter and maternal exercise (*p*_*lxe*_ < *0.001*).

**Table 3:** Effect of low intensity physical exercise training on metabolism and fat pad stores in offspring rats.

Parameters	NL-SM	SL-SM	SL-SM	NL-EM	Source of variation
Birth weight (g)	6.10 ± 0.02	6.14 ± 0.04	6.05 ± 0.01	6.10 ± 0.03	LxE^ns^ / E^*ns^ / L^ns^
Body weight (g) P21	47.0 ± 2.0	68.0 ± 1.7	46.9 ± 0.7	49.2 ± 1.3	LxE^*^ / E^**^ / L^****^
Body weight (g) P90	370. ± 6.6	406.6 ± 8.5	366.3 ± 3.9	374.2 ± 10.7	LxE ^ns^ / E^**^ /L^*^
Retroperitoneal fat pad (g/100 g) P90	1.248 ± 0.042	1.603 ± 0.099	1.09 ± 0.03	1.19 ± 0.113	LxE^ns^ / E^****^/ L^*^
Periepididymal fat pad (g/100 g) P90	1.14 ± 0.040	1.39 ± 0.091	0.97 ± 0.02	1.03 ± 0.091	LxE ^ns^ / E^***^/ L^*^
Mesenteric fat pad (g/100 g) P90	0.69 ± 0.030	1.06 ± 0.060	0.70 ± 0.03	0.73 ± 0.033	LxE^****^ / E^****^/ L^****^
Fasting Glucose (mg/dL) P90	83.57 ± 1.59	112.02 ± 1.68	97.08 ± 2.11	87.9 ± 2.46	LxE^****^ / E^****^/ L^****^
Fasting Insulin (ng/mL) P90	0.35 ± 0 01	0.54 ± 0.04	0. 25 ± 0.03	0.36 ± 0 05	LxE^ns^ / E^**^/ L^***^
HOMA-IR P90	1.86 ± 0.07	3.76 ± 0.30	1.37 ± 0.17	2.00 ± 0.29	LxE^**^ / E^****^/ L^****^

Data are expressed as the mean ± SEM (n = 6–18) at P0, P21 and P90. LxE, interaction between the exercise factor and the litter factor; E, exercise factor and L, litter factor; *p < 0.05, **p < 0.01, ***p < 0.001, ****p < *0.0001* and ns, no significant difference, based on a two-way analysis of variance.

Low intensity physical exercise during pregnancy and lactation mediated changes in the offspring’s fat pad stores in adulthood. The SL-SM rats exhibited higher weights of the retroperitoneal, periepididymal and mesenteric fat pad stores than the NL-SM rats (*p*_*l*_ < *0.05*, Table 3). Although the NL-EM rats only showed a 15% decrease in the periepididymal fat pad stores compared to that of the NL-SM rats, the SL-EM rats exhibited a lower weight in all evaluated fat pad stores when compared to that of the SL-SM rats, which demonstrated a significant effect of maternal exercise on the offspring fat depots (*p*_*e*_ < *0.0001*, Table 3).

### Long-term effects of low intensity physical exercise in pregnant and lactating dams on the glucose-insulin homeostasis of the adult offspring

At P90, SL-SM rats showed a higher fasting plasma glucose than NL-SM rats (*p*_*lxe*_ < *0.001*, Table 3), while SL-EM rats exhibited a 21.5% decrease compared to that of the SL-SM rats (*p*_*e*_ < *0.0001*; *p*_*lxe*_ < *0.0001*, Table 3).

**Figure 4:**
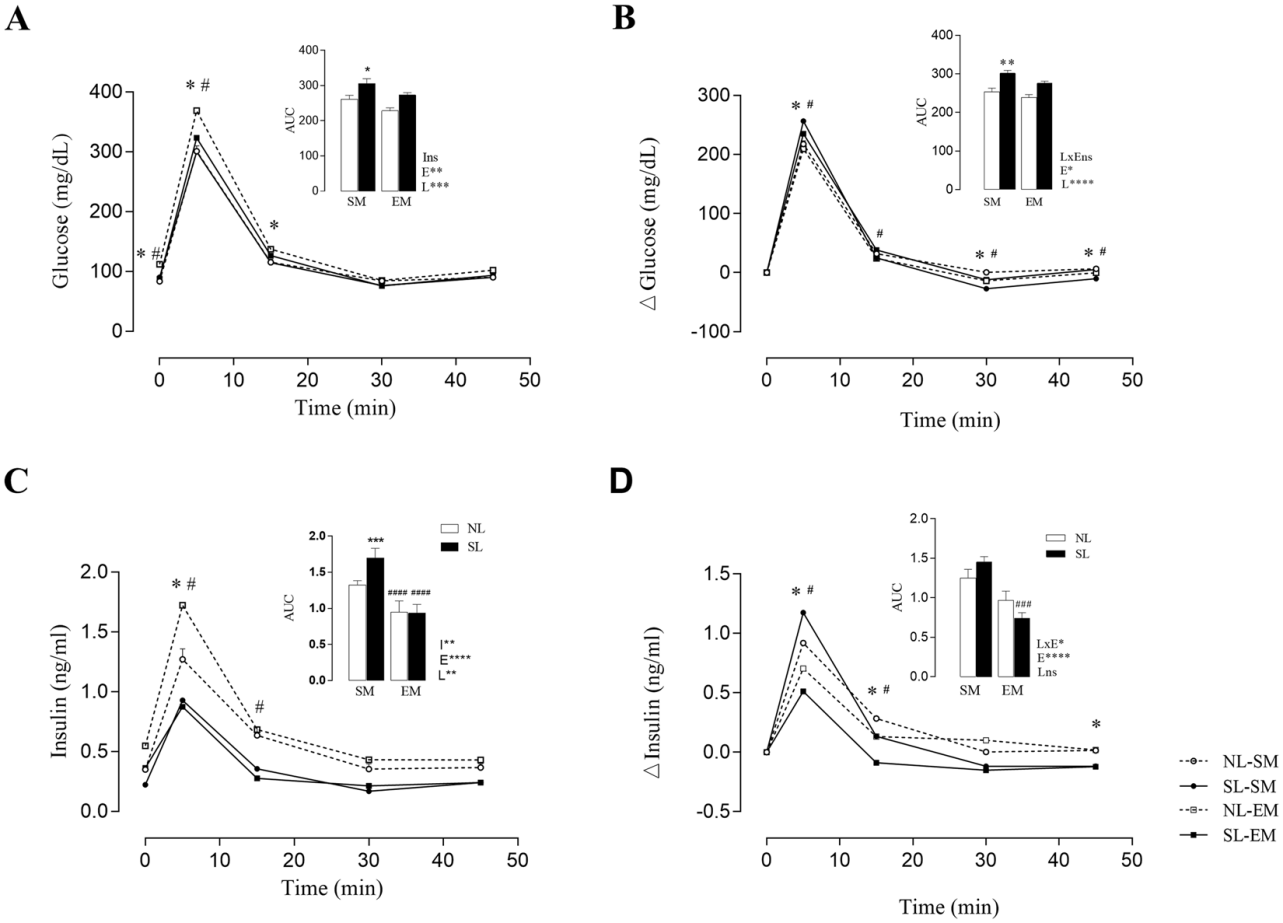
Effect of low intensity physical exercise training in adult offspring plasma glucose and insulin levels during the ivGTT. The upper panel represents the area under the curve (AUC). (**a**) plasma glucose, (**b**) plasma insulin, (**c**) increments (Δ) of plasma glucose and (**d**) increments (Δ) of plasma insulin. LxE, interaction between the exercise factor and the litter factor; E, exercise factor and L, litter factor; *p < 0.05 **p < *0.005* and ***p < 0.001, ****p < *0.0001* by two-way ANOVA and Tukey’s test. (n = 6–18). The ^#^ or * represents *p* < *0.05* in each time points of the bw curve, was calculated by repeated measures ANOVA. Over the lines and bars, (*) represents NL-SM compared with SL-SM and (^#^) represents SL-SM compared to SL-EM.

**Figure 5:**
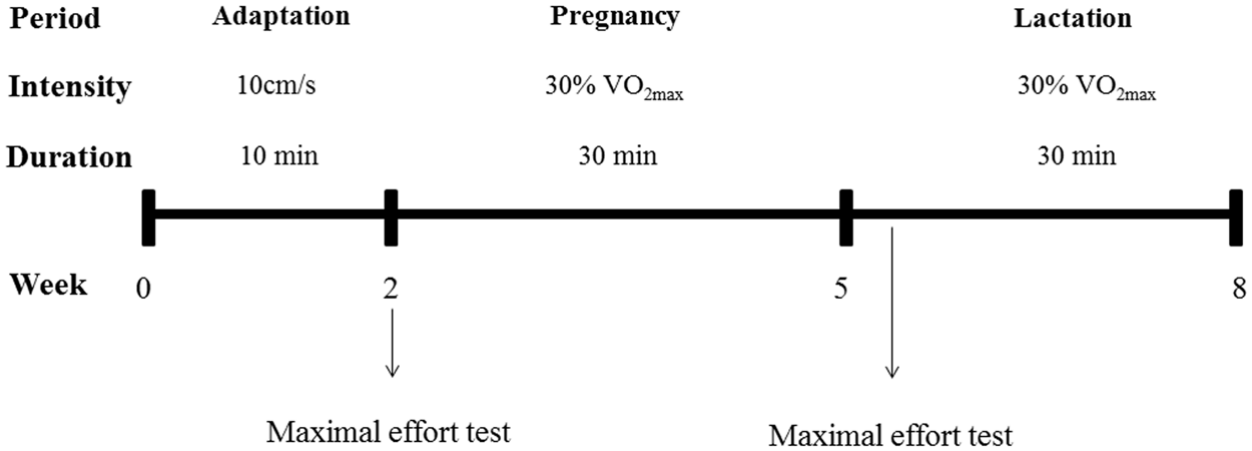
Low intensity physical training program in female rats during pregnancy and lactation periods, according to velocity, duration and intensity of sessions.

Glucose intolerance was detected in the SL-SM group rats, which exhibited alterations in plasma glucose levels during the ivGTT compared to NL-SM rats. In the Fig. 5a, during the ivGTT, plasma glucose, showed a significant higher levels at 0, 5 and 15 peak time point, 35% (*p* < *0.01*), 23% (*p* < *0.0001*) and 20% (*p* < *0.05*) respectively, as well as at 0 and 5 peak time point, the plasma glucose was 25% (*p* < *0.05*) and 12% (*p* < *0.01*) higher in SL-EM than NL-EM respectively. SL-SM rats, as shown by the 16.9% increase in the AUC, compared with NL-SM group (*p*_*l*_ < *0.005*, Fig. 4a). There was no significant difference in glucose levels between adult rat offspring from exercised mothers in relation to their counterpart control groups (Fig. 4a).

SL-SM group exhibited higher plasma insulin levels during the ivGTT compared to NL-SM rats. In the Fig. 5c, at 5 peak time point, 50% (*p* < *0.0001*), as well as at 5 and 15 peak time point, the plasma insulin was 31% (*p* < *0.0001*) and 49% (*p* < *0.001*) higher in SL-EM compared with LS-SM respectively. SL-SM rats, as shown by the 16.9% increase in the AUC, compared with NL-SM group (*p*_*l*_ < *0.005*, Fig. 4a). Similarly, insulin levels were increased during ivGTT in the SL-SM compared to SL-EM group. These animals showed a 50% increase in the AUC of the insulin plasma levels compared to SL-EM group (*p*_*l*_ < *0.001*, Fig. 4c).

During ivGTT, SL-SM plasma glucose and insulin increments (Δ), showed significant higher difference at 5, 15, 30 and 45 minutes peak time points of the plasma glucose, as well as in the 5, 15 and 45 minutes time point of the plasma insulin, compared to NL-SM (Fig. 4b,d). SL-EM plasma glucose and insulin increments, also showed significant difference at 5, 30 and 45 minutes peak time points of the plasma glucose, as well as in the 5 and 15 minutes time point of the plasma insulin, compared to SL-EM (Fig. 4b,d). The glucose increments (Δ), levels were increased during ivGTT in the SL-SM compared to NL-SM group. These animals showed a 20% increase in the AUC of the glucose compared to NL-SM group (*p*_*l*_ < *0.001*, Fig. 4b). The same results were found in SL-SM group that shows an increase of 50% in the AUC of the insulin plasma levels increments (Δ), compared with SL-EM group (*p*_*l*_ < *0.001*, Fig. 4d).

At P90, the SL-SM rats presented a 54.2% increase in fasting insulinemia compared with that of the NL-SM rats (*p*_*l*_ < *0.001*, Table 3). In relation to their counterparts, the NL-EM and SL-EM animals showed decreases in fasting insulin of 28.5% and 33.3%, respectively, indicating a not able effect of maternal exercise on insulin levels (*p*_*e*_ < *0.01*, Table 3). The HOMA–IR values of the SL-SM rats were increased by 102.0% when compared to that of the NL-SM rats (*p*_*l*_ < *0.0001*). In contrast, the NL-EM rats exhibited a 26.3% decrease in HOMA-IR values compared to that of the NL-SM rats (*p*_*lxe*_ < *0.01*, Table 3), and the values of the SL-EM rats were 46.8% lower than the values observed in their counterpart rats (*p*_*lxe*_ < *0.01*, Table 3). Altogether, the results showed a significant interaction between maternal exercise and small litter size.

## Discussion

The current study demonstrates for first time that maternal low intensity physical exercise during pregnancy and lactation was able to prevent obesity and metabolic dysfunction in adult male offspring exposed to early postnatal overnutrition. Small-litter offspring from exercised dams presented low depots of adipose tissue and low fasting insulin and glucose plasma levels, as well as normal glucose tolerance and insulin sensitivity. Interestingly, maternal low physical exercise improved maternal glucose metabolism and VO_2max_ capacity and enhanced sympathetic nerve electrical activity and increase insulin milk levels. Our results highlight the beneficial effects of low intensity maternal physical exercise on the health status of the offspring and mother.

Early overnutrition is an established model for the study of its long-term consequences in an animal model. Studies have shown that small litter size during the suckling period leads to overnutrition because of the reduced competition for milk and increase caloric intake^14^. Early-overfeeding has been shown to malprogram hypothalamic leptin resistance^15^, and reduce the thermogenic activity of brown adipose tissue^16^. Combined, these changes may well predispose individuals to exhibit hyperphagic behavior and adipose tissue accumulation due to lack of sympathetic-induced energy wastage. Interestingly, in the current study, mothers that performed low intensity physical exercise throughout pregnancy and lactation displayed reduced sympathetic nervous tone, and normoinsulinemia but elevated concentrations of insulin in their milk. We hypothesised that it may be the altered levels of insulin in the milk that contributed to attenuation of early-overfeeding induced obesity in their rat offspring.

Beyond the wellcharacterised action of insulin on food intake, body weight and energy balance in the hypothalamus^17^, insulin also regulates the function of several hypothalamic areas by modifying neuronal plasticity, especially during early life by promoting metabolic derangement and neuronal dysfunction associated with impaired synaptic plasticity^18^. Accordingly, it is possible that the offspring of physical exercise dams ingested more insulin via milk during the first 21 days of life and that this attenuated or modulated the early overfeeding effects in neuronal pathways involved in energy regulation and thermogenesis function in rat immature brain.

Postnatal early nutrition, especially breast-feeding, is essential to infant development, protecting against obesity and metabolic dysfunction in later life^19^. Studies have shown that human milk contains high concentrations of bioactive substances such as proteins, peptides, steroids, growth factors and hormones, including insulin^20–23^.

Oral insulin from the mother may function in the regulation of the growth and development of the neuroendocrine system, newborn immune system and gastrointestinal tract^24^. Additionally, insulin levels in milk appear to have a beneficial effect on gut maturation and prevent later diseases such as Crohn’s disease, celiac disease and type 1 diabetes^25–28^. Studies have documented the presence of insulin receptors in the mammalian intestine, in the jejunal and ileal brush border and intestinal crypt, in the fetal period, during the suckling period, at weaning, and in adults^20, 29–32^.

The macromolecule insulin is digested in the lumen of the gut to avoid absorption into the blood stream; however, there is some evidence that oral insulin treatment decreases bw, cholesterol and the triglyceride blood level in different animal models^22, 33^. In insulin-resistant states, the intestine significantly enhances the production of lipoproteins^34^ and glucose^35^. The mechanism by which luminal insulin influences intestinal metabolism even without being absorbed is not completely understood, but its capacity to downregulate gut insulin receptor expression^33^ might be the cornerstone factor.

Interestingly our study showed that exercised dams have a modified milk composition on the10^th^ and 21^st^ days of the lactation period with significantly higher levels of insulin. Studies have shown that exercise during lactation does not affect the quality of breast milk composition, however improves the maternal health condition^36–38^. Interestingly milk insulin levels during lactation was different in exercised mothers, where dams displayed high milk insulin levels, on the other hand, maternal low intensity physical exercise reduced plasma insulin. This result suggests a possible high skeletal muscle adaptation on uptake nutrients without insulin action. Physical exercise induces an increase in insulin sensitivity of tissues, such as the muscle^39^. The exact mechanisms that influence the hormone composition of milk are still unknown. However, we could suggest that insulin sensitivity in the mammary glands could be affected by physical exercise, and the increased insulin concentration in milk may be due to the different ways that this hormone acts in mammary glands of exercised mothers.

It is known that physical exercise training increases the peripheral insulin-sensitivity as a compensatory response to better uptake glucose for the physiological energy demand^39^. Insulin also plays a central role in protein milk synthesis^40–42^; in human beings, a decrease in insulin sensitivity in mothers results in a lower milk output in response to infant demand^43^.

Regular physical exercise during pregnancy improves maternal health conditions^44, 45^ that are important to fetal growth and development, which primarily depend on maternal placental transport for adequate fetal hormones, nutrients and oxygen supply to the fetus^46^. Low to moderate maternal exercise, approximately 40–65% of VO_2max_, during pregnancy has beneficial effects on offspring metabolism development in exercised mothers exposed to undernutrition^47^. The suggested mechanisms of these effects may be related to metabolic changes, promoted through blood flow and changes in the production of fetal and placental hormones that control development^48^.

Low intensity exercise (30% of the VO_2max_) during pregnancy and lactation was beneficial for maternal and offspring health. Mothers that were exposed to low intensity exercise, 30% VO_2max_, in the current study maintained a VO_2max_ after pregnancy similar to theVO_2max_ before pregnancy; on the other hand, the sedentary mothers showed a reduction in theVO_2max_ after pregnancy compared to their VO_2max_before pregnancy. The maintenance of theVO_2max_ may have resulted in improvements in placental growth and functional capacity. Aerobic exercise increases blood flow and provides better delivery of nutrients and oxygen, allowing for a better overall growth rate of the fetus in later pregnancy^49^. The improvement of the VO_2max_ may induce an increase in blood supply to tissues leading to high diffusion of oxygen, improving the ability to extract oxygen from the blood into all tissues^50^.

Physical exercise during pregnancy and lactation also contributed to a reduction in the fat pad stores and an improvement in glucose/insulin metabolism in dams. Exercised mothers exhibited a reduction in mesenteric adipose tissue stores and improved glucose metabolism associated with an increase in sympathetic electrical activity. Maternal exercise during pregnancy promotes autonomic nervous system balance and, consequently, has beneficial effects on brain function and structure in both the mother and her offspring in an animal model^51^. Studies have shown that the increase in parasympathetic nervous system activity and reduction in sympathetic nervous system activity contributes to obesity onset and insulin resistance in obese humans and animal models^52^. However, moderate physical exercise in adult male rats is able to improve the sympathetic nerve tone, enhance energy expenditure, and decrease fat stores and body weight^53^.

Interestingly, the improvement in the autonomic nervous system (ANS) activity is related to the VO_2max_ balance^54^. A sedentary lifestyle decreases the VO_2max_ and leads to the development of an increase in fat deposition and body weight gain in humans^55^. Studies have shown that the beneficial effect of physical exercise on metabolism in pregnant humans and animals is dependent on the type, intensity and frequency of exercise^13, 56^. The American College of Sports Medicine guidelines recommend 30 minutes or more of moderate exercise daily for pregnant women in the absence of medical or obstetric complications^57^. According to most protocols, the exercise is considered moderate when theVO_2max_ is between 50–70%^5^. High intensity exercise promotes a deleterious effects in pregnant mothers^11^ and subsequent generations^8^.

The ANS is involved in the fatty acid mobilization induced by physical exercise^58^. The activity of the heart is also stimulated by the SNS during exercise, which functions to increase blood flow, particularly to the muscles, improving nutrition^59, 60^, and adipose tissue, stimulating the lipolysis pathway^61, 62^. Previous studies from our group have shown that moderate physical exercise promotes a beneficial effect on glucose metabolism by improvement of pancreatic islet function and ANS activity in an adult obese animal model^63^ and induces a decrease in fat pad stores, related to activation of the sympathoadrenal axis^64, 65^. Furthermore, we found that mothers submitted to low intensity exercise show an improvement in ANS activity, suggesting a balance in glucose metabolism, and a decrease in mesenteric fat pad stores and increase in VO_2max_ compared to that of sedentary mothers after pregnancy. This may contribute to the improvement of maternal health, leading to the prevention of metabolic programming associated with overnutrition.

In conclusion, the current study suggests that maternal low intensity physical exercise during the perinatal period improves the health of the mother and prevents metabolic dysfunction in offspring later in life. These protections can be associated with changes in the maternal milk composition, including high insulin content, suggesting a potential reprogramming effect.

## Materials and Methods

### Ethical approval

The handling of animals and the experimental procedures were in accordance to the rules of the National Council of Animal Experiment Control (CONCEA) and the Brazilian Society of Science in Laboratory Animals (SBCAL) and approved by the Ethics Committee on Animal Use of Universidade Estadual de Maringa – CEUA/UEM (protocol number 9427151014).

### Animals

At 70 days of age, female *Wistar* rats were mated with 80-day-old male rats insets of 3:1, respectively. Pregnancy was confirmed by the presence of sperm cells in the vaginal plug (pregnancy day 0.5). The pregnant rats were maintained in individual cages and distributed into 2 groups: Exercised mothers (EM) and, sedentary mothers (SM). The exercise was performed throughout pregnancy and lactation. The maternal body weight (bw) was measured throughout the pregnancy and lactation periods.

At LD3, mothers were distributed into 4 groups: Exercised mothers with normal litter (EM-NL), Exercised mothers with small litter, (EM-SL), sedentary mothers with normal litter (SM-NL), and, sedentary mothers with small litter (SM-SL). At postnatal day 3 (P3) Offspring were distributed into 4 groups: Normal litter of exercised mothers (NL-EM), small litter of exercised mothers (SL-EM), normal litter of sedentary mothers (NL-SM) and small litter of sedentary mothers (SL-SM), and each lactating dam (4 litters for each experimental group) was housed with 9 pups (preferentially male). Considering the issue of sexual dimorphism and that litter size manipulation has been shown to have significant effects on male rats compared to females^66, 67^,we used only males in this study. However, when the required number of male offspring in the litter was not reached, females newborns were used to adjust the litter size to 9 pups throughout the sucking phase. To induce early overnutrition, on the third day after birth, the litter size was adjusted to 3 male pups per dam. The offspring were placed in an environmentally controlled room and received water and standard chow (Nuvital, Curitiba, Brazil) *ad libitum*.

### Exercise protocol

#### Adaptation and protocol for the maximal effort test

During the mating period (approximately 1 to 2 weeks), female rats were acclimated, using a modified protocol, to a treadmill for rats (Panlab, Harvard Apparatus^®^, Cornellà- Barcelona – Spain) 10 minutes per day, 3 times a week at 10 cm/s^68^. After detection of pregnancy, the animals were submitted to an effort test to determine the velocity of the training throughout pregnancy. On the third day of lactation, the second test was performed to determine the intensity of the exercise protocol (30% VO_2max_). The test was performed twice: on pregnancy day 0.5 and LD3 (the period of parturition), using a treadmill for rodents with an indirect calorimetry analyzer *(Panlab technology for bioresearch, Harvard Apparatus* ^*®*^ *- Le405, gas analyzer)* for the determination of the O_2_/CO_2_ gas concentrations.

The test began with a warm up (5 minutes, 10 cm/s, 0° of inclination), after which the velocity was increased by 5 cm/s every 3 minutes until the exhaustion of the animal^69^. The VO_2max_determination was used to calculate the intensity of the training (30%) for each phase of training, based on the maximal velocity (100% maximal effort) of the VO_2max_ test. Exercised and sedentary dams were submitted to the effort test at the same time to compare the physical performance.

#### Physical training protocol

The physical exercise training began 24 h after the effort test. On LD3, the animals performed an other effort test to adjust the speed training for the lactational period (30%). The training was performed three times a week, 30 minutes/day during pregnancy and the lactation period, using 30% of the maximal velocity obtained in the effort test (Fig. 5). We did not use an electrical stimulus to keep the animals running.

#### Body weight

Maternal bw (n = 10–12 per group) was measured throughout pregnancy and the lactation period. The bw of the offspring (n = 6–18 per group) was determined once weekly throughout the experimental protocol. The total area under the curve (AUC) for body weight was calculated^70^.

#### Milk sample collection

For milk samples collection, another batch of dams at LD10 and LD21 (n = 8–10 per group); lactating mothers were separated from their pups for 2 h before collection^71–73^. The fed dams were anesthetized with sodium thiopental (45 mg/kg of BW, i.p., Thiopentax®, Cristália, Itapira, São Paulo, Brazil) and received an injection (2.5 UI/kg of BW, i.p.) of synthetic oxytocin (Oxytocin®, Chemical Union, Embu, São Paulo, Brazil) to induce milk secretion^74, 75^. Breast milk samples were collected by manually massaging the nipple (0.5 ml/dam) and stored at −20 °C for subsequent analysis. Milk samples were diluted (1:20 v/v) in saline solution (0.9% NaCl) for glucose measurement using the enzymatic colorimetric glucose oxidase method with a commercial kit (Gold Analisa; Belo Horizonte, Minas Gerais, Brazil)^76^.

#### Lipid profile

Milk samples (n = 8–10 per group) were diluted (1:20 v/v) in saline solution (0.9% NaCl) for total cholesterol measurement using the enzymatic colorimetric cholesterol oxidase method with a commercial kit (Gold Analisa; Belo Horizonte, Minas Gerais, Brazil)^77^ and triglyceride concentration using the enzymatic colorimetric glycerol-3-phosphate oxidase method with a commercial kit (Gold Analisa; Belo Horizonte, Minas Gerais, Brazil)^78^.

#### Intravenous glucose tolerance test (ivGTT)

For the ivGTT, another batch of dams at LD21 (n = 5–6 per group), and offspring at P90 (n = 6–18 per group),underwent a surgical procedure under ketamine and xylazine anesthesia (3 and 0.6 mg/100 g of bw) to implant a silicone cannula into the right jugular vein for the ivGTT, as previously described^79^. Animals were allowed to recover 24 hours after surgery. Rats fasted for 12 hand were then infused with a glucose load (1 g/kg bw). Blood samples were obtained from the silicone canula 0, 5, 15, 30 and 45 minutes after glucose injection. Glucose and insulin levels were measured using biochemical analyses. The delta peak glucose in the ivGTT was calculated by the subtraction of the fasting plasma glucose and insulin concentration was used to obtain the glucose (Δglucose) and insulin changes (Δ insulin) for each time point of the ivGTT. Increases in total Δglucose and Δinsulin were calculated with the glucose and/or insulin AUC for the 45 minutes of the ivGTT^80^.

#### Radioimmunoassay and biochemical analyses

Plasma and milk insulin were measured by radioimmunoassay (RIA) in a gamma counter (Wizard2 Automatic Gamma Counter, TM-2470, PerkinElmer^®^, Shelton, CT, USA). Standard human insulin, and anti-rat insulin antibody (Sigma-Aldrich^®^, St. Louis, MO, USA), and ^125^I-labeled recombinant human insulin (PerkinElmer^®^, Shelton, CT, USA) were used. The intra-assay coefficients of variation were in the range of 8–10%. The limit of detection was 0.006 ng/ml. The plasma glucose, milk glucose, and lipid profile was determined by using a commercial kit (Gold Analisa^®^, Belo Horizonte, MG, Brazil)^81^.

#### Sympathetic and parasympathetic electrical activity assessment

For the autonomic nerve activity assessment at LD21, another batch of dams (n = 5–6 per group) fasted for 12 h and was subsequently anesthetized with thiopental (45 mg/kg bw); longitudinal incisions were made on the anterior cervical region under a dissection microscope to isolate the nerve bundle of the left superior branch of the vagus nerve from the carotid artery. The nerve was covered with silicone oil to prevent dehydration and placed on a pair of curved silver recording electrodes (0.6 mm diameter) connected to an electronic device (Bio-Amplificator, Insight®; Riberão Preto/SP, Brazil) that amplified the electrical signals up to 10,000 times, and the low and high frequencies, 1–80 kHz, were filtered. The neural signal output was acquired by an Insight interface (Insight®, Riberão Preto, Brazil), viewed online and stored by a personal computer running software (Bio-Amplificator, Insight®; Riberão Preto/SP, Brazil). For data acquisition, recordings took place in a Faraday cage to avoid any electromagnetic interference. Nerve activity was analyzed as the number of spikes/ 5 s (after a 2-minute period of signal stabilization), and 20 record frames of 15 s from each animal were randomly chosen for spike counting. The average number of spikes was used as the nerve firing rate for each rat. Also, the branch of the sympathetic nerve from the lumbar plexus (retroperitoneal white adipose tissue innervation - greater splanchnic nerve) was dissected. The electrode was placed under the greater splanchnic nerve, close to the retroperitoneal area. Firing rates from the nerve were obtained as described for the vagus nerve^68^.

#### Removal of fat pad stores for measurement

After the experimental procedures, dams (n = 10–12) and offspring (n = 6–18) were euthanized, and fat pad stores (mesenteric, retroperitoneal and periepididymal) were removed and weighed to assess the state of obesity. Fat pad store values were correlated with the rat bw and calculated as g/100 kg of bw^80^.

### Statistical analysis

Results were reported as means SEM. Statistical analysis and graphics were performed using GraphPad Prism^®^ version 6.01 for Windows (GraphPad Software, Inc. San Diego, CA, USA). Data sets were analyzed using Two-way analysis of variance (ANOVA) followed by Tukey’s *post hoc* test. *p* < *0.05* was considered significantly different when considering the main effect of exercise (E), litter size (L), their interaction (LxE; litter size vs exercise) and the differences between groups. Changes in body weight and ivGTT were analyzed using repeated measure ANOVA.
